# Effect of Application of a Homogeneous Magnetic Field During Chemical Crosslinking of Magnetic Collagen-Based Hydrogels with Genipin on Their Essential Properties

**DOI:** 10.3390/polym17182437

**Published:** 2025-09-09

**Authors:** Adriana Gilarska, Wojciech Horak, Agnieszka Radziszewska, Damian Rybicki, Czesław Kapusta

**Affiliations:** 1 Faculty of Physics and Applied Computer Science, AGH University of Krakow, Mickiewicza 30, 30-059 Krakow, Poland; 2 Faculty of Mechanical Engineering and Robotics, AGH University of Krakow, Mickiewicza 30, 30-059 Krakow, Poland; 3 Faculty of Metals Engineering and Industrial Computer Science, AGH University of Krakow, Mickiewicza 30, 30-059 Krakow, Poland

**Keywords:** collagen, SPIONs, magnetic hydrogels, static magnetic field, genipin

## Abstract

The aim of this study was to investigate the effect of a static, homogeneous magnetic field on the physicochemical properties of magnetic hydrogels based on collagen and superparamagnetic iron oxide nanoparticles (SPIONs), chemically crosslinked with genipin. The crosslinking process was initiated in the presence of a magnetic field with three different induction values (100, 250 and 500 mT), generated in specially designed experimental systems. It was demonstrated that the applied field did not noticeably affect the crosslinking efficiency, and stable hydrogels with a high gel fraction in the range of 87–94% were obtained. STEM image analysis revealed that in the highest magnetic field, the nanoparticles tended to form larger clusters, while at lower fields and in the material crosslinked at zero field, smaller clusters and chains of nanoparticles were observed mainly. This observation was reflected in the magnetic susceptibility, which showed a weaker response to the magnetic field of the material obtained by crosslinking in the presence of the 500 mT field compared to the material crosslinked without the field—larger clusters of nanoparticles may hinder the alignment of the magnetic moments of their constituent nanoparticles. Studies of the physicochemical properties of the hydrogels obtained indicate that the presence of larger clusters can cause a local decrease in the crosslinking density, resulting in a slight decrease in the storage modulus and increased initial swelling and degradation rates. The results obtained show that the application of a homogeneous magnetic field with moderate induction values during the crosslinking process can be used as a tool for modification of the microstructure of magnetic collagen-based hydrogels. The possibility of such structural modifications may be useful in designing biomaterials with properties tailored to their target application.

## 1. Introduction

Collagen is a structural protein that is the main component of the extracellular matrix of most mammalian connective tissues, found, e.g., in bones, cartilage and skin. Its main function is to ensure the stability and structural integrity of tissues, but it also influences cell adhesion, growth and differentiation through interaction with specific receptors [1]. A characteristic structural feature of collagen proteins is the formation of a right-hand helix, which constitutes a single collagen fibril molecule. The helix is formed by three left-hand polypeptide chains twisted around their own axis. Collagen is synthesised in the extracellular space, and this process is multi-stage and involves the formation of tropocollagen molecules, which form microfibrils and then fibrils, which ultimately assemble into mature collagen fibres [2]. Collagen is an attractive biomaterial for a wide range of applications in regenerative medicine, both in the construction of artificial organs and in tissue engineering. It is considered one of the most important materials in connective tissue regeneration due to its hydrophilicity, excellent biocompatibility, minimal inflammatory response, elasticity and biodegradability [3]. The use of collagen is focused on applications in bone and skin regeneration and wound healing [4]. Collagen-based hydrogels are very popular in tissue engineering [5,6]. The main limitation to their use as scaffolds is their rapid degradation and poor mechanical properties [7]. An effective approach to minimising these disadvantages is the use of various crosslinking methods [8]. In addition, enriching the collagen network with additional inorganic components to create hybrid materials is also an attractive solution. Such a combination can enhance the physicochemical and biological properties of hybrid materials compared to those of the polymer matrix alone [9,10].

In recent years, magnetic hydrogels containing magnetic nanoparticles (mainly iron oxides) have attracted growing interest, including systems based on collagen as a natural biopolymer [11]. The presence of magnetic nanoparticles allows remote control of the material using an external magnetic field, which opens up new possibilities for the design of smart biomaterials that respond to physical stimuli [12]. Magnetic hydrogel analogues in form of systems of functionalized magnetic nanoparticles, can be used in drug delivery [13,14], targeting cell growth in tissue engineering [15], and cancer therapies such as hyperthermia [16,17] or circulating tumour cell capture [18]. In the context of the above applications, the research focuses mainly on the use of a magnetic field to a stable material after the crosslinking process, to control drug transport, to influence cell activity to raise the local temperature or to separate circulating tumour cells. However, another aspect that may be significant in the design of hydrogel materials with magnetic properties is the fact that in the presence of a magnetic field, nanoparticles can be oriented and aligned, which affects their spatial distribution and can potentially influence the structure of the gel forming. From the point of view of biomedical applications, particularly in tissue engineering, the possibility of spatial ordering of material structures opens a promising approach to obtain gradient or anisotropic materials that could better reflect the architecture of natural tissues [19,20] or lead to obtaining new structures. The approaches to nanoparticle ordering described in the literature and the associated possibility of collagen fibre orientation usually take place in the process of fibrillogenesis during incubation at 37 °C. Shi et al. incorporated SiO_2_@Fe_3_O_4_ rods into a collagen hydrogel and, under the influence of a static magnetic field, obtained composites with parallel or perpendicular alignment of the magnetic rods to the gel surface [21]. Antman-Passig et al. demonstrated that a magnetic field orders spherical magnetic nanoparticles in a collagen-based hydrogel, aggregating them into chains along the field lines [22]. Similar results were obtained by Wright et al., where magnetic anisotropic collagen hydrogel promoted the orientation of human adipose stem cells parallel to the direction of the magnetic nanoparticles and collagen fibres, which can be used in tendon repair [23].

In contrast to the magnetic ordering of the structure during fibrillogenesis under physiological conditions, this work presents preliminary results of studies on the effect of a static magnetic field with different induction values on the essential physicochemical properties of collagen hydrogels containing superparamagnetic iron oxide nanoparticles during chemical crosslinking with genipin. Genipin is one of the most effective and biocompatible crosslinking agents, reacting with primary amine groups present in the collagen structure, leading to the formation of a covalent network and ensuring the structural stability of collagen hydrogels, which is desirable for tissue engineering applications [24]. In this work, chemical crosslinking of collagen-based hydrogels doped with magnetic nanoparticles (SPIONs) was performed with genipin in the presence of a static magnetic field with the aim to verify if it is possible to control the forming structure with the application of a magnetic field. The magnetic systems were specially designed to ensure that the magnetic field acting in the hydrogel space was homogeneous, facilitating control over the uniform interaction with the whole volume of the reacting material. The essential parameters of the obtained hydrogels, such as microstructure, rheological and magnetic properties, gel fraction, swelling and degradation, were analysed to assess if the magnetic field can influence the forming structure at the chemical crosslinking stage.

## 2. Results and Discussion

### 2.1. Experimental Systems Generating a Homogeneous Magnetic Field

In order to carry out studies on the effect of a magnetic field on the structure of a magnetic hydrogel forming during chemical crosslinking, attention was paid to obtaining systems generating static magnetic fields with relatively high homogeneity to ensure better control and repeatability of the experimental conditions. In contrast to a gradient field, the homogeneous field does not generate an additional force causing the nanoparticles to move towards the higher field intensity but only affects the orientation of their magnetic moments and influences their dipolar interactions [25]. Our magnetic experimental systems were designed for use in the simultaneous chemical crosslinking of hydrogels, and therefore, in addition to the physical phenomena associated with the magnetic field, the effects associated with the forming network had also to be taken into account. The homogeneity of the field should therefore allow for the elimination of the effect of additional variables that hinder interpretation and allow carrying out preliminary studies, the results of which can then be used for the study of more complex systems.

**Figure 1:**
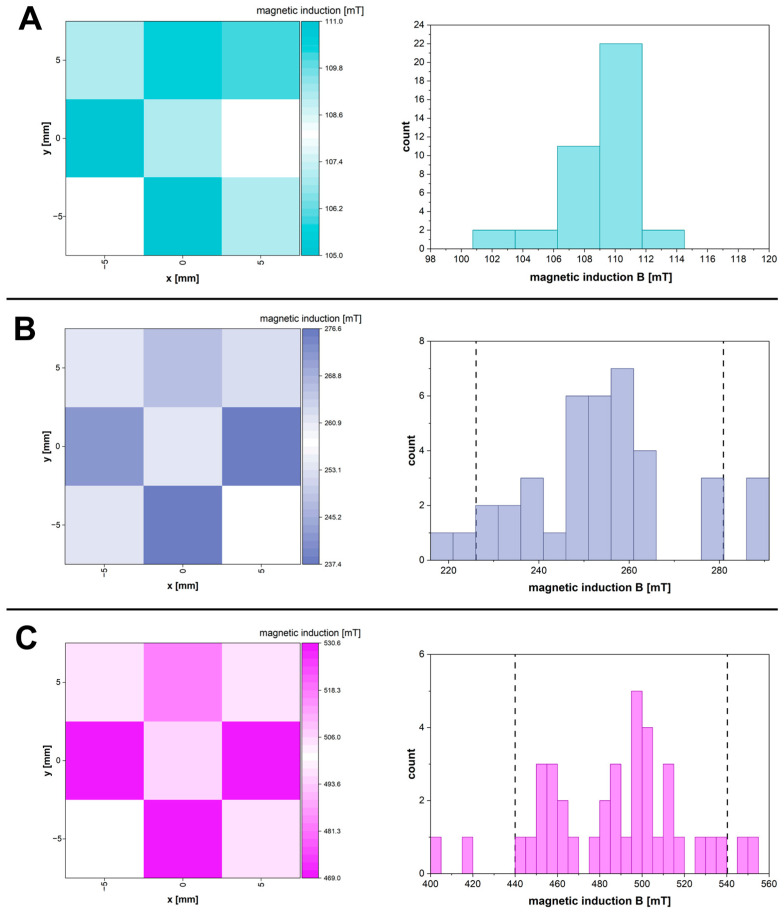
Heat maps for area of central plane of sample (**left**) and histograms (**right**) showing distributions of magnetic field induction generated by the constructed neodymium magnet systems with target values of 100 mT (**A**), 250 mT (**B**) and 500 mT (**C**). The areas on the histograms between the vertical dotted lines contain measurements within 10% of the average value (**B**,**C**).

Three separate neodymium permanent magnet systems were designed and constructed to generate a static, homogeneous magnetic field with target magnetic induction values of 100 mT, 250 mT and 500 mT (i.e., 1, 2.5 and 5 kOe field strength, respectively). The choice of magnetic field intensity considered potential applications requiring exposure of a human body to it. The selected field values are within the range of moderate magnetic fields achievable and commonly used in biomedical applications. Previous studies have demonstrated the safety and effectiveness of such fields in many areas, including bone regeneration, wound healing and stem cell differentiation [26,27]. Furthermore, systems generating moderate-intensity magnetic fields are easier to implement in experimental conditions compared to those generating higher fields, which may be an advantage in the context of future applications [27]. In order to assess the magnetic field distribution in the sample area (cylindrical shape, diameter 13 mm, height 5 mm), magnetic induction (*B*) measurements were performed for each of the three planes: the base (bottom), the middle section and the top surface of the sample, located between the magnets (sample planes parallel to the field lines). In each plane, magnetic induction measurements were performed at the centre of the plane and the points towards the edges. Table 1 shows the average values of the magnetic field induction for the designed systems, obtained for all the measurement points from the three planes of the sample area. Figure 1 shows heat maps for the central cross-section of the sample area and histograms for all the measurement points (for all three planes) for the three constructed magnetic systems. The highest homogeneity was obtained in the system generating the field with the lowest intensity—for all the measurement points the values were within 6% of the average value. In the case of systems generating fields with higher induction, the values for the majority of points were within 10% of the average value (marked with dotted lines on the histograms in Figure 1B,C). Despite the presence of a few larger deviations from the average, the field distribution in the main volume of the sample was relatively uniform for systems with medium and highest field induction values, which was considered satisfactory to classify the conditions as homogeneous in the context of further experiments. For better clarity and ease of interpretation of the results, the target magnetic field values (100, 250 and 500 mT) assumed in the magnetic systems designing were used in the rest of the paper and in the sample descriptions.

### 2.2. Preparation of Magnetic Hydrogels

**Figure 2:**
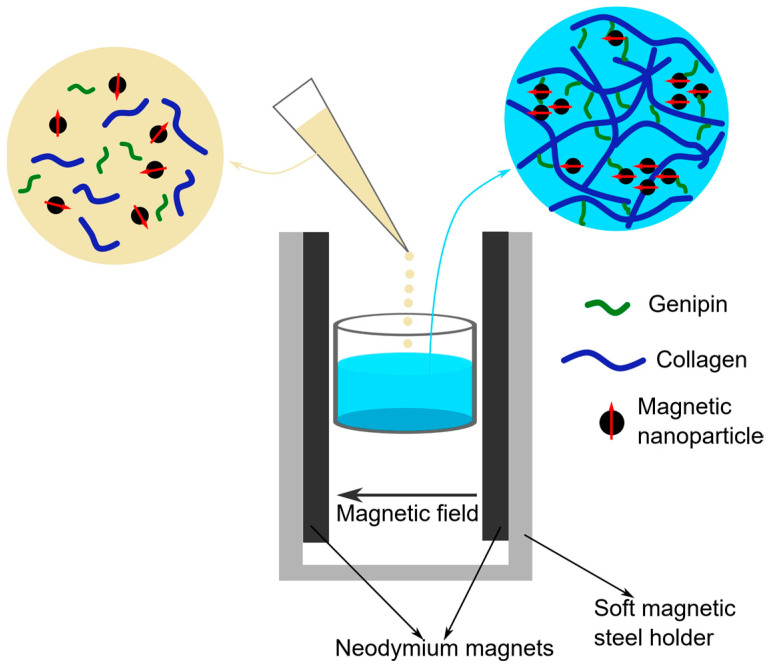
Schematic diagram of the process of crosslinking collagen hydrogel with genipin in a designed dedicated magnetic system. Immediately after adding genipin, the polymer sol was transferred to a cylindrical sample form and left for 30 min.

Collagen-based magnetic hydrogels were obtained by introducing an aqueous suspension of superparamagnetic iron oxide nanoparticles (SPIONs) coated with a cationic chitosan derivative into a collagen sol, followed by the introduction of a crosslinking agent, genipin, and placement of the prepared mixture into magnet systems generating a homogeneous field (Figure 2). The magnetic nanoparticles synthesised according to a previously developed procedure [28] were characterised by a magnetic core with a size of approximately 15 nm, and the cationic chitosan-based coatings ensuring their stability in aqueous suspension. In the previous study, we demonstrated an effective method for obtaining magnetic hydrogels based on chitosan and collagen, in which both biopolymers and magnetic nanoparticles participate in chemical crosslinking with genipin, which reacts with amino groups present in the collagen, chitosan and chitosan-coated nanoparticles [29]. This approach allowed us to obtain a structurally stable nanocomposite with uniformly embedded magnetic nanoparticles. In the present work, we focused on obtaining a magnetic collagen-based hydrogel in which the chemical crosslinking process is performed in the presence of a static, homogeneous magnetic field. SPIONs coated with a cationic chitosan derivative can therefore not only participate in the crosslinking process and incorporate themselves into collagen fibres (structural stability), but also, due to the magnetic field action, orient themselves and influence the microstructural changes in the forming fibrous collagen network, which could potentially allow the structure of the forming hydrogel to be adjusted as needed.

Three types of materials named Col100, Col250 and Col500 were obtained, and their crosslinking was performed in designed systems generating a static magnetic field of three target induction values (100, 250 and 500 mT, respectively). A hydrogel crosslinked without the application of a magnetic field (Col0) was obtained as a control. All the samples contained the same amounts of collagen and magnetic nanoparticles, and the same concentration of genipin was used. The crosslinking process was carried out at room temperature in order to focus on the potential influence of the field on the forming hydrogel. During the crosslinking process, no visual differences in the forming network were observed between the samples. All the samples transitioned from a transparent sol state to a preliminary gel after the first 30 min of crosslinking in a magnetic field, and then to a well-formed hydrogel after 24 h of crosslinking, with a milky colour and a slightly purple glow.

### 2.3. Gel Fraction

The gel fraction was determined by immersing dried hydrogels in water for 24 h, then drying them again and comparing the dry weights of the hydrogels before and after incubation in water. The results of the gel fraction test are presented in Table 2. High gel fraction values were observed in all samples, ranging from 87 to 94%, regardless of the presence and the value of the magnetic field induction. No significant differences were observed between the groups, and no trend suggesting the influence of the field on the degree of hydrogel crosslinking was observed. These results indicate that the application of a magnetic field during the first 30 min of the crosslinking with genipin did not affect the chemical crosslinking process of the collagen-based hydrogel with magnetic nanoparticles. The high gel fraction values obtained mean that most of the material was effectively crosslinked and only a small portion was washed out. It can therefore be concluded that in the experimental systems used, the presence of a magnetic field and its effect on magnetic nanoparticles in the collagen network does not interfere with the crosslinking process on a macroscopic scale.

### 2.4. Microstructure (STEM)

**Figure 3:**
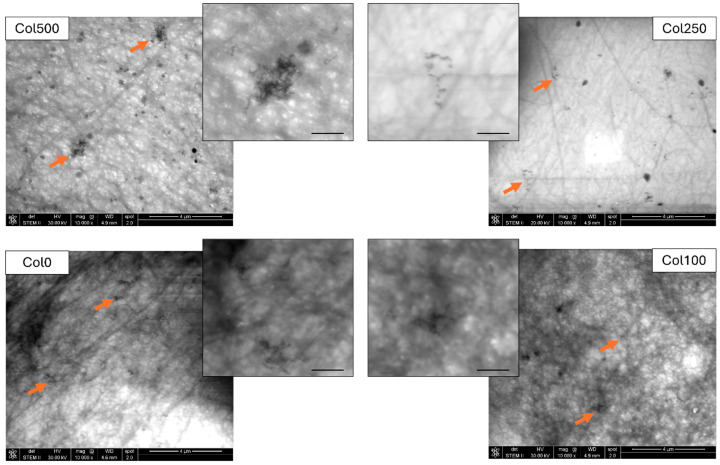
STEM images of the materials obtained. Orange arrows indicate selected chains and clusters of magnetic nanoparticles, the scale bars on the zoomed images represent 500 nm.

The microstructure of the magnetic hydrogels obtained was analysed with a scanning transmission electron microscope (STEM). The STEM images of the magnetic hydrogels are presented in Figure 3. Imaging such hydrogel structures with STEM is challenging due to the properties of the material and the specific way of sample preparation on the measuring grid. A gel layer that is too thick on the grid may prevent the detection of structural details, including magnetic nanoparticles, while a structure that is too thin carries the risk of burning out during scanning. Despite these difficulties, we were able to obtain images of all the analysed samples in a quality that allowed the identification of nanoparticles in the collagen matrix and the comparison of their arrangement depending on the crosslinking conditions. In our previous work [29], we showed that superparamagnetic iron oxide nanoparticles coated with a cationic chitosan derivative tend to form irregular chains that integrate into the structure of collagen-chitosan hydrogel structure, with the nanoparticles tending to arrange themselves along collagen fibres and a higher collagen content in the sample resulted in a tendency to form shorter chains of magnetic nanoparticles. In this work, a similar tendency was observed for collagen hydrogels crosslinked without the use of a magnetic field (Col0) and for hydrogels crosslinked under the influence of lower field intensities (Col100 and Col250). In the collagen structure of these hydrogels, magnetic nanoparticles can be seen forming shorter or longer chains, some of which are branched. A slightly different trend was observed in the structure of the hydrogel crosslinked in the presence of a field with the highest magnetic induction value (Col500). Here, chains of nanoparticles were clearly visible, forming much larger clusters in the structure compared to the other samples. To explain these differences, it is necessary to consider which phenomena may occur when nanoparticles interact with a static homogeneous magnetic field. Nanoparticles exhibiting superparamagnetism in the presence of a magnetic field become magnetised, reaching significantly higher values of magnetisation compared to known biological structures, and their magnetisation is not permanent and disappears when the magnetic field is switched off. For this reason, they are advantageous structures in biomedical applications [30]. When a homogeneous field is applied, the magnetic moments of the nanoparticles can align themselves in the direction of the field lines. The absence of a field gradient means that no force acting on the magnetic nanoparticles causes them to move; however, the orientation of the magnetic moments can lead to the formation of chains of nanoparticles, as the nanoparticles can then attract each other along the field lines through dipolar interactions, analogously to small magnets (for the superparamagnetic nanoparticles these interactions are induced only in the presence of a field; without a field, the effect disappears) [31]. The formation of chains of nanoparticles is not a clear indication of the presence of a magnetic field, as such structures have been observed in collagen-chitosan hydrogels crosslinked without a field [29], and in the present work, a hydrogel based on collagen alone crosslinked at no field also contained such chains. The formation of chains is typical for magnetic nanoparticles coated with a cationic chitosan derivative [32,33]. This may be caused by the interaction of the coating itself (being a cationic chitosan derivative), diffusion during gel formation, and reaction with genipin. However, in the sample crosslinked in the presence of the field with the highest induction value (Col500), a tendency to form larger clusters of nanoparticles was observed. This may indicate that such a field enhances the orientation effect of nanoparticles owing to a more effective quenching of the superparamagnetic fluctuations and their dipole interactions are stronger. They then acquire magnetic moments large enough to promote the formation of larger magnetic structures in the hydrogel network [31]. This observation was also reflected in the differential magnetic susceptibility, which is discussed in the next section. At the same time, such local orientation of magnetic nanoparticles did not interfere with the overall efficiency of the chemical crosslinking process. The possibility of influencing the distribution and orientation of nanoparticles in the collagen hydrogel structure during the chemical crosslinking process with genipin by a homogeneous field offers the potential to control the local properties of the hydrogel network or to obtain a higher local magnetic moment.

### 2.5. Magnetic Properties (VSM)

**Figure 4:**
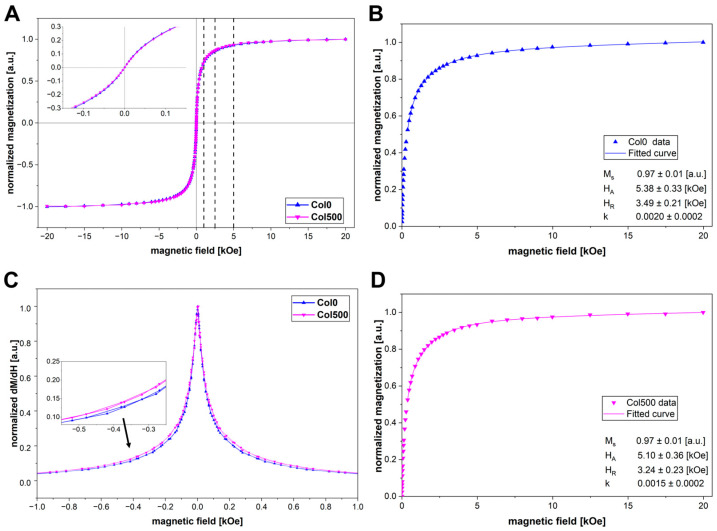
Normalised magnetization curves for Col0 and Col500 samples measured at 300 K (**A**), normalised magnetization curve together with a fit according to Equation (1) for Col0 and Col500 in panels (**B**) and (**D**), respectively. *M_s_* is the saturation magnetization, *H_A_* is the anisotropy field, *H_R_* is the correlation field. Normalised differential susceptibility in function of the magnetic field for Col0 and Col500 samples (**C**). The vertical dashed lines in (**A**) mark the magnetization values at magnetic fields of 1, 2.5 and 5 kOe, respectively. The inset graph in (**A**) shows a zoomed area at small fields region. The inset graph in (**C**) shows a zoomed area of the differential susceptibility curves.

The magnetic properties of magnetic hydrogels were analysed at a temperature of 300 K using a vibrating sample magnetometer. Taking into account the observations in the STEM images, two types of samples were analysed for their magnetic properties: a hydrogel crosslinked without a field (Col0) and a hydrogel crosslinked in the presence of field with the highest induction (Col500). The dependence of magnetisation on the magnetic field for the obtained hydrogels is shown in Figure 4A. As expected, the magnetisation curves for both materials are similar, with zero coercivity, which confirms that the nanoparticles in the systems studied retain their superparamagnetic properties. In magnetic fields with values of 1, 2.5 and 5 kOe (corresponding, respectively, to 100, 250 and 500 mT of the magnetic field induction generated by the designed systems), the magnetisation of magnetic nanoparticles reached 74%, 86% and 93% of the saturation magnetisation, respectively, indicating effective quenching of the superparamagnetic fluctuations and rather low magnetic anisotropy of the nanoparticles. In order to obtain quantitative information regarding the magnetic anisotropy a so-called law of approach to saturation can be used to fit the *M*(*H*) dependence. Here, we used a relevant model proposed by Safronov et al. [34]:

(1) $$M\left(H\right)=M_{s}\left(1-\frac{H_{A}^{2}}{15H^{0.5}\left(H^{1.5}+H_{R}^{1.5}\right)}\right)+kH$$

where *M_s_* is the saturation magnetization, *H_A_* is the anisotropy field, *H_R_* is the correlation field and term *kH* stems from the possible response of the nanoparticle shell. We found that this model can be used to reliably describe *M*(*H*) dependence starting from about 40–50% of the saturation magnetization. Figure 4B,D shows the results of such a fit for both samples. The obtained anisotropy field *H_A_* is the same within the error margin for both samples and rather small compared to, e.g., the nanoparticles used in the original work by Safronov et al. [34].

In order to investigate in more detail potential changes in the distribution and orientation of magnetic nanoparticles in the hydrogel structure, the differential magnetic susceptibility was analysed (Figure 4C), which allows for a deeper insight into the dynamics of the magnetic response in the material [35]. The differential magnetic susceptibility curves for Col0 and Col500 are similar in shape, with a pronounced peak at the origin, but the curve for the sample crosslinked without a field (Col0) is narrower than for the sample crosslinked in a 500 mT field (Col500). This indicates that in the sample crosslinked without a field, the magnetic response of the nanoparticles is stronger, allowing them to orient themselves more easily along the field [35,36]. In the case of Col500 hydrogel, the response to the magnetic field is weaker, which may be related to the presence of larger clusters that may hinder the alignment of the magnetic moments of nanoparticles grouped around each other. This observation is consistent with the larger clusters of nanoparticles observed in STEM images for Col500 compared to the smaller chains of nanoparticles in Col0.

### 2.6. Rheological Properties

**Figure 5:**
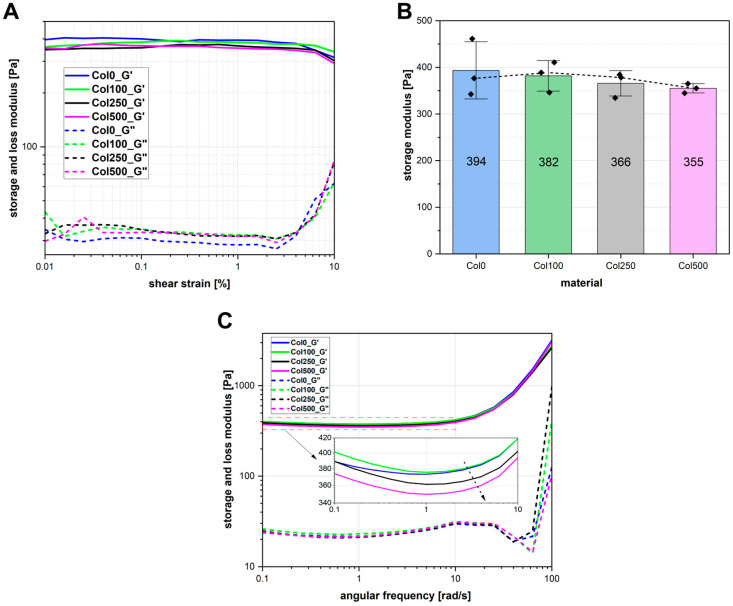
Amplitude sweep (**A**), the values of storage modulus for γ = 1% (**B**) and frequency sweep (**C**) analyses for magnetic hydrogels obtained. In (**B**), black dots represent measured G′ values, and the dotted line represents the line connecting the middle values of the results, inset in (**C**) is a zoomed area from 0.1 to 10 [rad/s].

The rheological properties of the magnetic hydrogels were analysed by performing amplitude sweep and frequency sweep tests. Figure 5A shows the averaged results of the amplitude sweep measurement, which were used to determine the linear viscoelastic range (LVE). All tested samples exhibited good structural stability, with the LVE range extending to at least γ = 2%. Therefore, γ = 1% was selected for the frequency sweep analysis to ensure measurements were conducted within the LVE region.

To evaluate the stiffness of the samples, the storage modulus (G′) was analysed (Figure 5B). The hydrogel crosslinked in the absence of a magnetic field (Col0) showed the highest average stiffness (394 Pa), and with increasing magnetic field intensity during crosslinking, the value of G′ decreases. The lowest G′ was recorded for the sample crosslinked in the field with the highest induction—Col500 (355 Pa). Although the total difference in G′ values was approximately 10%, a consistent trend was noted: higher magnetic field induction during crosslinking resulted in a reduction in the elastic properties of the hydrogel network. Additionally, the variability of G′ differed among the samples, with Col0 exhibiting the highest standard deviation and Col500 the lowest.

Frequency sweep analysis (Figure 5C) confirmed that all tested samples exhibited gel-like properties, with a clear dominance of the elastic component (G′ > G″) across the entire frequency range. All the samples showed a relatively stable, high storage modulus at low frequencies. A rapid increase at higher frequencies is typical for polymer structures. A slight reduction in G′ in the mid-frequency range may indicate the occurrence of relaxation processes in the tested hydrogel matrices. Overall, the results suggest that the materials exhibited a stable internal structure and resistance to deformation over a wide frequency range. This confirms that the crosslinking process was effective both in the absence and in the presence of a magnetic field. The highest stiffness was observed in the hydrogel crosslinked without a field (Col0) and the hydrogel crosslinked at the lowest magnetic field induction (Col100), while Col500 exhibits the lowest G′ values, which means a slightly less developed gel structure.

Taking into account that chitosan coatings of magnetic nanoparticles may participate in the genipin crosslinking process, differences in the distribution of nanoparticles in the hydrogel network structure could have affected the local network density, which could have caused slight differences in stiffness between the samples. The literature describes systems in which, at a constant nanoparticle concentration, an increase in particle size results in a decrease in the elastic modulus of the hydrogel material [37]. Nanoparticles forming larger clusters in the Col500 sample may have less surface area available for interaction with collagen fibres during crosslinking compared to more separated, single chains. Local network disturbances may therefore occur in the neighbourhood of nanoparticle clusters [38,39], but this does not cause a significant deterioration in elastic properties. Furthermore, it is worth noting that the spread in results is greatest for the Col0 sample, while the Col500 sample, despite its lowest stiffness, shows the least variation (Figure 5B). The higher standard deviation of the values measured for a given type of hydrogel may be due to the presence of heterogeneity in the samples, resulting from differences in the crosslinking density in different areas of the gel [40]. This may indicate that in the Col500 sample, the dominant mechanism for nanoparticle distribution is their orientation in clusters, resulting from dipole interactions. Clusters may form locally, but their distribution throughout the sample volume may be more uniform than the more random distribution of smaller clusters and single chains in samples crosslinked without a magnetic field and at the presence of fields with lower induction (100 and 250 mT).

Rheological studies have shown that the application of a constant uniform magnetic field during the crosslinking of collagen hydrogel containing magnetic nanoparticles can induce local structural modification. Despite these changes, the macroscopic crosslinking efficiency remained high, as confirmed by gel fraction analysis. These findings suggest that magnetic fields may serve as a supplementary tool for fine-tuning the microstructural homogeneity of magnetic hydrogels, with minimal compromise to their mechanical integrity. Such control could be beneficial in the development of biomaterials with consistent and predictable mechanical behaviour.

### 2.7. Swelling and Degradation

**Figure 6:**
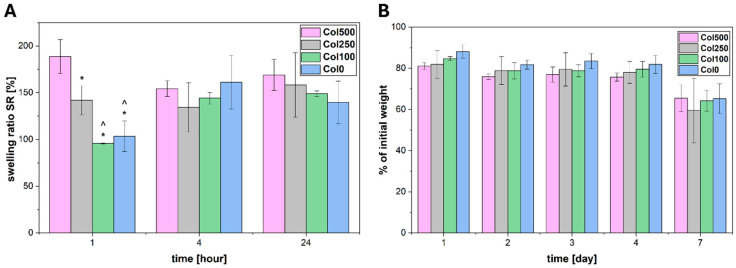
Swelling ratio (**A**) and weight loss (**B**) for magnetic hydrogels incubated in PBS buffer. In (**A**) * indicates statistical significance when compared with Col500 after 1 h (*p* < 0.05), ^ indicates statistical significance when compared with Col250 after 1 h (*p* < 0.05).

Swelling and degradation of the obtained magnetic hydrogels were carried out in PBS buffer at 37 °C. The swelling capacity was tested for 24 h, and degradation was tested for 7 days. The results are presented in Figure 6. In the case of swelling (Figure 6A), the greatest differences between the tested hydrogels are visible after the first hour of the experiment. The highest swelling capacity in the initial phase of the experiment was demonstrated by the hydrogel crosslinked in the highest magnetic field (Col500) (statistically significant difference). Based on the analysis of STEM images and rheological properties, such swelling after the first hour of incubation in PBS may confirm a local reduction in crosslinkage density resulting from the presence of larger clusters of nanoparticles, which favoured faster solution penetration. According to rubber elastic theory and rheological measurement data, the crosslinking density for Col0, Col100, Col250 and Col500 reached 0.153 ± 0.024 mol/m^3^, 0.148 ± 0.013 mol/m^3^, 0.142 ± 0.010 mol/m^3^ and 0.138 ± 0.004 mol/m^3^, respectively. The differences are statistically insignificant, but the Col500 had the tendency to show the lowest crosslinking density, which may contribute to its increased swelling after the first hour. Subsequently, a slight decrease was observed for this hydrogel after 4 h, which could be the result of the loss of weaker network fragments, followed by relative stabilisation after 24 h. The lowest swelling was observed for the hydrogel crosslinked without a field (Col0) and the hydrogel crosslinked in the lowest field (Col100) after the first hour of the experiment. After 4 and 24 h, no significant differences were observed between all the materials tested, suggesting that the influence of the magnetic field-induced modification was limited mainly to the initial phase of the process, during which differences resulting from local changes in the microstructure may be accentuated, and this effect disappears after reaching equilibrium in water absorption capacity.

Degradation tests performed in PBS (Figure 6B) showed that all tested hydrogels maintained good stability under the analysed conditions. During the first four days of the experiment, the weight remained within the range of 75–88% of the initial weight, with no significant changes between the first and fourth days. After seven days, more noticeably decreased weights were observed, reaching values in the range of 60–65% of the initial weight. In the initial period (4 days), a general trend was observed that with an increase in the magnetic field induction used during the crosslinking process, the degree of degradation increased slightly, but these differences were not statistically significant. After the seventh day, all the samples of hydrogels tested retained a similar percentage of their initial weight. The trend observed can be correlated with the swelling test results—the sample crosslinked in the highest magnetic field showed a greater initial swelling capacity, which may indicate the presence of more free space in the gel structure at the regions of nanoparticle clusters. Such a structure could have promoted both faster fluid absorption and easier degradation at the initial stages of the test [41].

## 3. Summary and Conclusions

This paper presents results of the studies on the essential physicochemical properties of magnetic hydrogels based on collagen and superparamagnetic iron oxide nanoparticles, in which chemical crosslinking with genipin was performed in a static, homogeneous magnetic field of three different induction values, generated in specially designed and constructed systems. The results obtained show that the magnetic field did not noticeably affect the efficiency of the crosslinking process—structurally stable hydrogels were obtained in all samples. Simultaneously, the interaction of magnetic nanoparticles with the magnetic field caused local differences in the structure of the material. STEM imaging revealed that in the highest field used, the nanoparticles tend to form larger clusters distributed throughout the hydrogel volume, while samples crosslinked at lower fields and without a field were characterised by smaller clusters or single chains of nanoparticles. The effect of formation of larger clusters in samples crosslinked in the highest field was found to be consistent with the results of magnetic characterisation. The analysis of rheological properties, swelling and degradation indicated that the presence of such aggregates could lead to local changes in the crosslinking density, resulting in a slight decrease in the storage modulus and increased swelling and degradation rates at the initial time interval of the swelling and degradation tests, due to the lower network density in the areas of nanoparticle clusters.

The results obtained indicate that a static, homogeneous magnetic field with a suitably chosen induction value can locally affect the structure of a collagen-based magnetic hydrogel with magnetic nanoparticles during its formation at chemical crosslinking with genipin. In the next stage, research can therefore be undertaken on the selection of proper chemical crosslinking parameters, the concentration of magnetic nanoparticles, as well as tests with cell culture in order to obtain materials with a specific structural order for designing scaffolds that are both stable under physiological conditions and suited to the implantation method and site.

## 4. Materials and Methods

### 4.1. Materials

Collagen type I from rat tail (3.14 mg/mL solution, Corning, NY, USA), genipin (98%, Challenge Bioproducts Co., Douliu City, Taiwan), iron(III) chloride hexahydrate and iron(II) chloride tetrahydrate (Sigma-Aldrich, St. Louis, MO, USA), ammonia (25% solution, Chempur, Piekary Śląskie, Poland), cationic chitosan derivative (obtained according to the procedure developed earlier [42]).

### 4.2. Preparation of Systems Generating a Static, Homogeneous Magnetic Field

To construct systems generating a homogeneous magnetic field, appropriate combinations of neodymium magnets (single magnets with a diameter of 30 mm and a thickness of 2 mm) were selected in order to obtain a static magnetic field with target induction values of 100, 250 and 500 mT between the magnets in the sample area. The sample area (cylindrical shape, diameter 13 mm, height 5 mm) was located in the centre between the magnets. In order to measure the values of the magnetic field induction in the sample volume, three planes were chosen (base, middle section and top surface of the sample area). Magnetic field induction measurements (Smart Magnetic Sensor Type SMS-102, Tel-Atomic Incorparated, Jackson, MI, USA) were performed for each plane at 13 points arranged in a regular grid every 3.25 mm, covering the central point of the sample and points in the radial direction to its edges, respectively.

### 4.3. Details of Preparation of Magnetic Hydrogels

In order to obtain magnetic hydrogels, 110 μL of an aqueous suspension of superparamagnetic iron oxide nanoparticles (SPIONs) coated with a cationic chitosan derivative (iron content 235 μg/mL) was added to a collagen solution (0.74 mL). The nanoparticles were synthesised according to a previously developed procedure [28]. Next, 150 μL of genipin (10 mM, solution in 10× PBS) was added to the magnetic sol. After vigorous shaking of the sol, it was introduced into forms (internal dimensions: 13 mm diameter, 5 mm height) placed between magnets in the constructed magnetic systems. The materials were left in the magnetic systems for 30 min of the main stage of crosslinking, after which the crosslinking process was completed at room temperature for 24 h. This resulted in the formation of Col100, Col250 and Col500 magnetic hydrogels, for which the crosslinking process was performed in a magnetic field with an induction of 100, 250 and 500 mT, respectively. As a control, Col0 hydrogel was obtained under the same conditions (time, temperature), but without the application of a magnetic field.

### 4.4. Gel Fraction Determination

The gel fraction was determined by incubating dry hydrogels in water for 24 h at room temperature and then drying them again. The hydrogels were air-dried until their weight stabilised. The gel fraction was determined using the following formula:

(2) $$gel\;fraction\;\left[\%\right]=\frac{W_{d\_final}}{W_{d\_initial}}\cdot 100$$

where $W_{d\_initial}$ represents the dry weight of the hydrogel before incubation in water, and $W_{d\_final}$ represents the dry weight of the hydrogel after incubation in water.

### 4.5. Microstructure Imaging

The microstructure of the materials was imaged using a scanning transmission electron microscope (STEM, FEI Nova NanoSEM 450, FEI Company, Hillsboro, OR, USA). For this purpose, a drop of biopolymer sol with nanoparticles was placed on a copper grid and incubated according to the hydrogel preparation procedure.

### 4.6. Vibrating Sample Magnetometry (VSM)

Determination of the magnetic properties of materials was performed using a vibrating sample magnetometer (VSM) option of a Quantum Design Physical Property Measurement System (PPMS-9, Quantum Design, San Diego, CA, USA) equipped with a superconducting 9 T magnet. Hysteresis loops have been measured at 300 K and magnetic field strength ranging from −80 to +80 kOe.

### 4.7. Rheological Characterisation

The rheological properties were tested using a MCR 302e rheometer (Anton Paar, Graz, Austria) equipped with a P-PTD200 + H-PTD200 measuring cell, which ensured high temperature stability during the measurement. The tests were performed at a temperature of 37 °C using a plate-plate measuring geometry (plate diameter d = 13 mm, measuring gap 2.5 mm). Two types of oscillatory tests were performed. Initially, an amplitude sweep test was carried out to determine the linear viscoelastic region (LVR), using a strain range of γ = 0.01–10% (logarithmic ramp) at a constant angular frequency of ω = 10 rad/s. Subsequently, a frequency sweep test was conducted within the LVR, applying a constant strain of γ = 1% and varying the angular frequency in the range ω = 0.1 to 100 rad/s (logarithmic ramp).

The rheological measurement data were also used to evaluate the crosslinking density of the hydrogels obtained (*n_e_*, mol/m^3^). It was calculated using the following formula [43]:

(3) $$n_{e}=\frac{G_{e}}{RT}$$

where *G_e_* represents plateau value of the storage modulus, *R* is the universal gas constant (8.314 J/(mol K)) and *T* is the measurement temperature (310 K).

### 4.8. Swelling Properties

Swelling was studied by incubating dried hydrogels in PBS buffer at 37 °C for 24 h. The swollen hydrogels were weighed after 1, 4 and 24 h of incubation. The degree of swelling (*SR*) at a given time point was calculated using the formula:

(4) $$SR\left[\%\right]=\frac{W_{s}-W_{d}}{W_{d}}\cdot 100$$

where *W_s_* represents the weight of the swollen hydrogel and *W_d_* represents the weight of the dry hydrogel.

### 4.9. Degradation Studies

Degradation was studied by incubating hydrogels (in a wet state) in PBS buffer at 37 °C with gentle shaking for 7 days. At specific time points (after 1, 2, 3, 4 and 7 days), the hydrogels were weighted, and the PBS was replaced with fresh PBS. The weight loss was calculated using the following formula:

(5) $$\%\;of\;initial\;weight=\frac{W_{t}}{W_{0}}\cdot 100$$

where *W*_0_ represents the initial weight of the hydrogel and *W_t_* represents the weight of the hydrogel after a specified incubation time in PBS.

### 4.10. Statistical Analysis

Experiments were repeated three times and results expressed as a mean ± standard deviation. Statistical significance was calculated using analysis of variance (ANOVA). A comparison between two means was analysed using Tukey’s test with statistical significance level set at *p* < 0.05.

## Figures and Tables

**Table 1:** Target and measured values of magnetic field induction in the designed magnetic systems (along with their standard deviations within the sample area).

	Target Value of Magnetic Field Induction *B* [mT]	Measured Value of Magnetic Field Induction *B* with Standard Deviation [mT]
system 1	100	109 ± 2
system 2	250	253 ± 17
system 3	500	486 ± 33

**Table 2:** Gel fraction for the materials obtained.

Material	Gel Fraction [%]
Col500	93.4 ± 4.0
Col250	94.6 ± 3.0
Col100	87.6 ± 6.1
Col0	92.5 ± 5.2

## Data Availability

The original contributions presented in this study are included in the article. Further inquiries can be directed to the corresponding author.
